# O-GlcNAc transferase congenital disorder of glycosylation (OGT-CDG): Potential mechanistic targets revealed by evaluating the OGT interactome

**DOI:** 10.1016/j.jbc.2024.107599

**Published:** 2024-07-24

**Authors:** Johnathan M. Mayfield, Naomi L. Hitefield, Ignacy Czajewski, Lotte Vanhye, Laura Holden, Eva Morava, Daan M.F. van Aalten, Lance Wells

**Affiliations:** 1 Department of Biochemistry and Molecular Biology, Complex Carbohydrate Research Center, University of Georgia, Athens, Georgia, USA; 2 School of Life Sciences, University of Dundee, Dundee, UK; 3 Department of Clinical Genomics and Department of Laboratory Medicine and Pathology, Mayo Clinic, Rochester, Minnesota, USA; 4 Department of Molecular Biology and Genetics, Aarhus University, Aarhus, Denmark

**Keywords:** neurodevelopment, O-linked N-acetylglucosamine (O-GlcNAc), O-GlcNAc transferase (OGT), transcription, transcription regulation, histone modification, O-GlcNAcylation, post-translational modification (PTM), protein-protein interaction, intellectual disability

## Abstract

O-GlcNAc transferase (OGT) is the sole enzyme responsible for the post-translational modification of O-GlcNAc on thousands of target nucleocytoplasmic proteins. To date, nine variants of OGT that segregate with OGT Congenital Disorder of Glycosylation (OGT-CDG) have been reported and characterized. Numerous additional variants have been associated with OGT-CDG, some of which are currently undergoing investigation. This disorder primarily presents with global developmental delay and intellectual disability (ID), alongside other variable neurological features and subtle facial dysmorphisms in patients. Several hypotheses aim to explain the etiology of OGT-CDG, with a prominent hypothesis attributing the pathophysiology of OGT-CDG to mutations segregating with this disorder disrupting the OGT interactome. The OGT interactome consists of thousands of proteins, including substrates as well as interactors that require noncatalytic functions of OGT. A key aim in the field is to identify which interactors and substrates contribute to the primarily neural-specific phenotype of OGT-CDG. In this review, we will discuss the heterogenous phenotypic features of OGT-CDG seen clinically, the variable biochemical effects of mutations associated with OGT-CDG, and the use of animal models to understand this disorder. Furthermore, we will discuss how previously identified OGT interactors causal for ID provide mechanistic targets for investigation that could explain the dysregulated gene expression seen in OGT-CDG models. Identifying shared or unique altered pathways impacted in OGT-CDG patients will provide a better understanding of the disorder as well as potential therapeutic targets.

OGT is the sole enzyme responsible for the dynamic posttranslational modification of β-linked *N*-acetyl-D-glucosamine (O-GlcNAc) onto serine and threonine residues of thousands of nucleocytoplasmic proteins (1, 2, 3, 4, 5). Conversely, O-GlcNAcase (OGA) is responsible for the removal of this modification (6). In humans, *OGT* resides on the X chromosome (7). Based on the human exome database, *OGT* is one of the most conserved genes in the human genome and is the most conserved gene encoding a glycosyltransferase (8, 9). OGT shares 73% homology in its amino acid sequence with its *Drosophila melanogaster* ortholog encoded by the polycomb group (PcG) gene *super sex combs* (*sxc*) (7, 10). In mammals, knockout of *OGT* is embryonic lethal (7). Mammalian cells require OGT, and recent studies have begun to elucidate why OGT is essential (11). Minimum levels of glycosylation are required to maintain cell viability, and noncatalytic functions of OGT are crucial in normal proliferation (8). Specifically, knockout of OGT in mESCs results in increased proteasome activity raising the level of free amino acids, which in turn causes hyperactivation of mTOR, a sensor responsive to amino acid levels, resulting in mitochondrial dysfunction and cell death (12). Beyond glycosyltransferase activity and noncatalytic functions, OGT is also the protease responsible for host cell factor 1 (HCFC1) maturation (13, 14, 15, 16), although this function is not required for cell survival (8). For normal function, the transcriptional regulator HCFC1 must be proteolytically cleaved into N-terminal and C-terminal fragments with OGT being responsible for this in mammals and Taspase 1 being responsible in *D. melanogaster* (14, 15, 17). Proper HCFC1 maturation is required for regulating cell cycle progression and proliferation as well as gene expression (18, 19).

OGT has 13.5 N-terminal tetratricopeptide repeats (TPR), and this TPR domain is responsible for multiple protein-protein interactions, including substrate selection (Fig. 1*A*) (20). The C-terminal catalytic domain of OGT is a CAzy glycosyltransferase family 41 domain (21) and has an intervening domain with possible roles in the regulation of OGT and a proposed phosphatidylinositol (3, 4, 5)-trisphosphate (PIP_3_) binding domain (22, 23, 24, 25). O-GlcNAcylation is involved in numerous biological processes, and increasing evidence suggests a crucial role for OGT in the regulation of multiple aspects of transcription, signaling, and embryonic development (26, 27, 28). This review will summarize our current understanding of variants of OGT causal for the intellectual disability (ID) symptoms seen in patients with OGT-CDG as well as discuss interactors of OGT that are causal for disorders with ID phenotypes. These interactors, often with known O-GlcNAc sites (Fig. 2), provide targets for future investigation that could elucidate molecular mechanisms underpinning the neurodevelopmental phenotype seen in patients with OGT-CDG.

**Figure 1:**
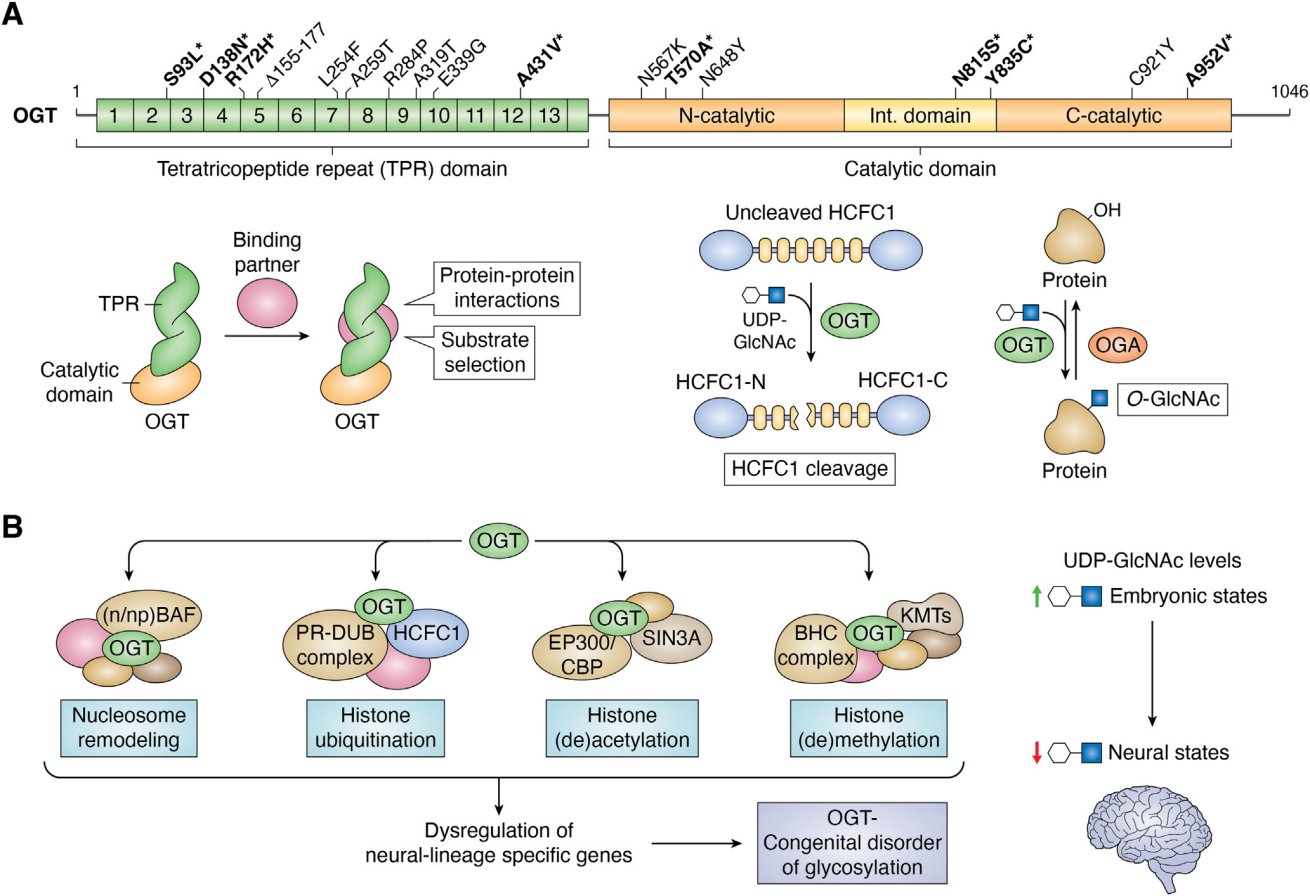
**Model figure: OGT structure, function, and possible mechanisms for OGT-CDG.***A*, domain map of OGT: OGT structure consists of both a N-terminal Tetratricopeptide repeat (TPR) domain (*green*) and a C-terminal catalytic domain (*orange*). The TPR domain of the nucleocytoplasmic isoform of OGT is composed of 13.5 degenerate 34 amino acid repeats and is involved in protein-protein interactions and substrate selection. The catalytic domain is responsible for the catalytic functions of OGT: HCFC1 cleavage and the addition of the O-GlcNAc modification onto substrate proteins. This domain contains both N-terminal and C-terminal catalytic regions linked by an intervening domain of unknown function (*yellow*). The functions of OGT are shown below the domain map. Depicted along the structure of OGT is a selection of the known variants of the protein, with those bolded and with an asterisk in the process of being characterized. *B*, the model this review proposes through which OGT is implicated in OGT-CDG: OGT interacts with and glycosylates components of complexes important in embryonic and neural development. This model suggests alterations in these OGT: protein interactions and hypoglycosylation of substrate proteins lead to dysregulation of gene expression and in part OGT-CDG phenotypes. Levels of UDP-GlcNAc in embryonic (high O-GlcNAc) and neural states (low O-GlcNAc) are essential for proper neurodevelopment, and this difference in substrate levels could explain the phenotype specificity of the disorder. *Pink* indicates proteins that interact with OGT but do not necessarily have known O-GlcNAc sites, termed here as binding partners. BHC, BRAF-HDAC complex; CBP, CREB-binding protein; EP300, Histone acetyltransferase p300; HCFC1, Host Cell Factor 1; Int. domain, intervening domain; KMTs, lysine methyltransferases; (n/np) BAF, neural/neuronal progenitor ATP-dependent BRG1/BRM associated factor; OGT, O-GlcNAc transferase; OGA, O-GlcNAcase; PR-DUB, Polycomb repressive deubiquitinase; TPR, tetratricopeptide repeat; UDP-GlcNAc, uridine diphosphate *N*-acetylglucosamine.

**Figure 2:**
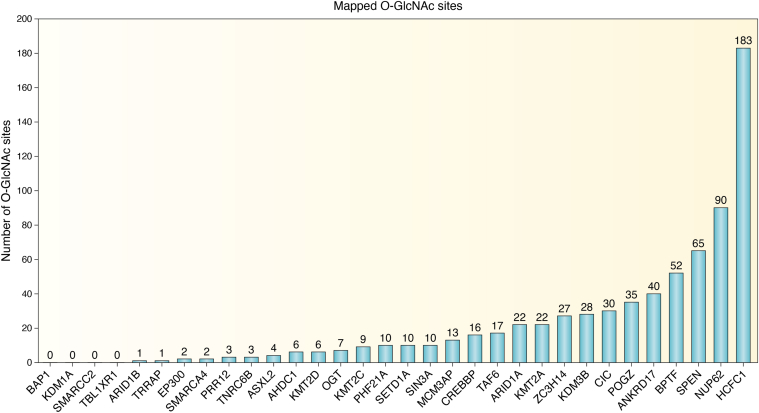
**Number of O-GlcNAc sites on identified OGT interactors**. Shown are OGT interactors identified by Stephen, *et al.* 2020 (60) that are catalogued by the Online Mendelian Inheritance in Man (OMIM) database to have an intellectual disability (ID) phenotype. The number of mapped O-GlcNAc sites for each interactor was obtained from The O-GlcNAc Database (5) from the Medical College of Wisconsin. Interactors are ordered from least to most mapped O-GlcNAc sites. Zero mapped O-GlcNAc sites do not confirm a protein is not O-GlcNAc modified by OGT, instead that particular protein may have a low O-GlcNAc site occupancy, may be sub-stoichiometrically modified by OGT, may have cell-type-specific modification, or the sites were not mapped at the time of the manuscript preparation.

To date, nine variants of OGT have been established as causal for OGT-CDG. The initial variants characterized were restricted to the TPR domain of OGT (OGT^L254F^, OGT^A259T^, OGT^R284P^, OGT^A319T^, OGT^E339G^, and OGT^Δ155-177^) (29, 30, 31, 32). However, variants in the catalytic domain of OGT have since been characterized and linked to OGT-CDG (OGT^N567K^, OGT^N648Y^, and OGT^C921Y^), confirming causal variants for this disorder are not domain-specific but span the entire protein (33, 34, 35). Characterization of additional variants in both the TPR and catalytic domains of OGT is ongoing (Fig. 1*A*, **bold∗**). The phenotypic presentation of the patients, the biochemical characterization of the variants, and the use of animal models will be discussed, highlighting the variable nature of OGT-CDG in all three aspects. Currently, many unanswered questions remain regarding OGT-CDG. As more pathogenic OGT variants are characterized and additional patients are identified, it may become possible to identify a set of common alterations in OGT function as well as a core set of clinical manifestations of OGT-CDG, which would aid in the diagnosis of the disorder.

## The OGT-CDG phenotype is variable

OGT-CDG patients present with a common set of core phenotypes but heterogenous secondary characteristics (Table 1). All patients exhibit core phenotypes of ID and developmental delay, often impacting their speech and language development. Common secondary features observed in patients with OGT-CDG include behavioral abnormalities such as Autism Spectrum Disorder (ASD) and Attention-Deficit/Hyperactivity Disorder (ADHD). Patients may present with brain abnormalities including microcephaly, eye abnormalities such as astigmatism, or dysmorphic features. Dysmorphic features vary from patient to patient but include coarse facial features, clinodactyly, or a dolichocephalic head. More variably presenting features include intense drooling, open mouth, hypotonia, and epilepsy or seizures. Finally, some patients affected by OGT-CDG present with more systemic phenotypes including connective tissue disorders, genital abnormalities, or ulcerative colitis. Overall, we see a high penetrance of neurological features; therefore, studies of OGT-CDG have focused on disease models relevant to neuronal development. These variants of OGT may also affect other organs or tissues given the heterogeneous nature of the clinical phenotypes present in patients with OGT-CDG. Additionally, OGT^A319T^ co-segregates with a mutation in the *MED12* gene, and OGT^L254F^ patient phenotypes vary within three generations of the same family (29, 31). These results suggest that genetic and environmental background may be influencing the molecular consequences of individual mutations.

**Table 1:** Identified OGT interactors linked to ID and their highlighted phenotypes and examples of known ID variants

Gene ID (MIM #)	Protein name	Alternative names	UniProt accession	Highlighted features of neurodevelopmental disorder	Example ID variants	References
**BAP1 (****619762****)**	**Ubiquitin carboxyl-terminal hydrolase BAP1**	**BRCA1 associated protein 1**	**Q92560**	**ID, developmental delay speech delay, motor delay, hypotonia, ASD/ADHD, dysmorphic facial features, eye abnormalities, skeletal malformations, growth failure**	**P12T, P12A, E31K, L49P, C91S, H169R, R718Q**	(341)
KDM1A (616728)	Lysine-specific histone demethylase 1A	LSD1, BHC110	O60341	ID, developmental delay, speech delay, growth failure, clinodactyly, craniofacial dysmorphisms, hypotonia, eye abnormalities, thin corpus callosum	E403K, D580G, Y785H	(95, 115)
SMARCC2 (618362)	SWI/SNF complex subunit SMARCC2	BAF170	Q8TAQ2	ID, speech delay, hypotonia, dysmorphic facial features, eye abnormalities, behavioral problems	Splice site, W241X, L610P, M896V	(342, 343, 344)
TBL1XR1 (616944, 602342)	F-box-like/WD repeat-containing protein TBL1XR1	TBLR1	Q9BZK7	ID, delayed psychomotor development, dysmorphic facial features, speech delay, behavioral problems	G70D, L282P, Y245C, frameshift	(345, 346, 347, 348)
				Developmental delay, dysmorphic facial features, ear abnormalities, eye abnormalities, speech delay, hypotonia	Y446C, Y445H, C325Y	(349, 350, 351, 352)
ARID1B (135900)	AT-rich interactive domain-containing protein 1B	BAF250B	Q8NFD5	ID, dysmorphic facial features, ear abnormalities, eye abnormalities, delayed psychomotor development, speech delay, seizures, hypotonia, brain abnormalities, behavioral problems	Frameshift and nonsense	(353, 354, 355, 356, 357)
TRRAP (618364)	Transformation/transcription domain-associated protein	Tra1 homolog	Q9Y4A5	ID, developmental delay, dysmorphic facial features, ear abnormalities, eye abnormalities, fifth finger clinodactyly, hypotonia, seizures, brain abnormalities, behavioral problems	R1986Q, L805F, A1043T, W1866R, W1866C	(358, 359)
EP300 (618333, 613684)	Histone acetyltransferase p300	P300, KAT3B	Q09472	ID, developmental delay, dysmorphic facial features, ear abnormalities, eye abnormalities, speech delay, behavioral problems	Q1824P, deletion	(360)
				ID, dysmorphic facial features, ear abnormalities, eye abnormalities, delayed psychomotor development, hypotonia, ASD, behavioral problems	Frameshift, termination, F1595V, N1286S	(361, 362, 363, 364, 365, 366)
SMARCA4 (614609)	Transcription activator BRG1	BRG1, BAF190	P51532	ID, dysmorphic facial features, ear abnormalities, eye abnormalities, delayed psychomotor development, hypotonia, absence of speech, brain abnormalities	Deletion, T859M, R885C, L921F, M1011T, R1157G	(355)
**PRR12 (****619539****)**	**Proline-rich protein 12**	**KIAA1205**	**Q9ULL5**	**ID, developmental delay, Motor delay, speech delay, ASD/ADHD, Eye abnormalities, hypotonia, growth failure, microcephaly, fifth finger clinodactyly**	**Frameshift, nonsense, splice site, R1169W, L1970P**	(367, 368, 369)
**TNRC6B (****619243****)**	**Trinucleotide repeat-containing gene 6B protein**		**Q9UPQ9**	**ID, developmental delay, dysmorphic facial features, ear abnormalities, eye abnormalities, delayed motor skills, speech delay, hypotonia, behavioral problems**	**Termination, frameshift, splice site, V1357E**	(370, 371)
ASXL2 (617190)	Putative polycomb group protein ASXL2		Q76L83	ID, dysmorphic facial features, ear abnormalities, eye abnormalities, hypotonia, delayed speech, delayed psychomotor development, seizures, brain abnormalities, behavioral problems	Frameshift	(372)
AHDC1 (615829)	AT-hook DNA-binding motif-containing protein 1	Gibbin, XIGIS	Q5TGY3	ID, dysmorphic facial features, ear abnormalities, eye abnormalities, hypotonia, delayed psychomotor development, brain abnormalities, behavioral problems	Frameshift, D1457G	(373, 374, 375, 376)
KMT2D (147920)	Histone-lysine specific N-methyltransferase 2D	MLL2	O14686	ID, developmental delay, dysmorphic facial features, ear abnormalities, eye abnormalities, short fifth finger, seizures, hypotonia	Termination	(377)
OGT (300997)	UDP-N-acetylglucosamine–peptide N-acetylglucosaminyl transferase 110 kDa subunit		O15294	ID, developmental delay, speech delay, Behavioral problems, hypotonia, eye abnormalities, ear abnormalities, brain abnormalities (microcephaly, thin corpus callosum), dysmorphic features (clinodactyly), epilepsy, seizures	L254F, A259T, R284P, A319T, E339G, N567K, N648Y, Δ155–177, N806S, S93L, A431V	(30, 31, 32, 33, 34, 35, 98, 378, 379)
KMT2C (617768)	Histone-lysine N-methyltransferase 2C	MLL3	Q8NEZ4	ID, dysmorphic facial features, ear abnormalities, eye abnormalities, hypotonia, delayed psychomotor development, speech delay, seizures, behavioral problems	Termination, frameshift	(380, 381)
PHF21A (618725)	PHD finger protein 21A	BHC80	Q96BD5	ID, developmental delay, dysmorphic facial features, clinodactyly, impaired motor skills, seizures, hypotonia, behavioral problems	Frameshift, termination, G429S	(116, 382)
**SETD1A (****619056****)**	**Histone-lysine N-methyltransferase SETD1A**	**KMT2F, SET1A**	**O15047**	**ID, developmental delay, dysmorphic facial features, ear abnormalities, eye abnormalities, speech delay, seizures, behavioral problems**	**Splice site, Y1499D, frameshift**	(92, 383)
SIN3A (613406)	Paired amphipathic helix protein Sin3a		Q96ST3	ID, developmental delay, dysmorphic facial features, ear abnormalities, eye abnormalities, clinodactyly, hypotonia, brain abnormalities, behavioral problems	Frameshift, termination	(182, 384)
MCM3AP (618124)	Germinal-center associated nuclear protein	GANP	O60318	ID, developmental regression, dysmorphic facial features, hypotonia, speech delay, delayed motor development, developmental regression, seizures	E915K, V1272M, frameshift, termination, R878H, S951P, L870S	(163, 385, 386, 387, 388)
CREBBP (618332, 180849)	CREB-binding protein	CBP, KAT3A	Q92793	ID, developmental delay, dysmorphic facial features, ear abnormalities, eye abnormalities, fifth finger clinodactyly, speech delay, seizures, behavioral problems	C1710R, R1867Q, R1868W, M1872V, E1724K	(389, 390)
				ID, dysmorphic facial features, ear abnormalities, eye abnormalities, fifth finger clinodactyly, brain abnormalities, speech delay, seizures, hypotonia, behavioral problems	Termination, R1378P, splicing	(391, 392, 393)
TAF6 (617126)	Transcription initiation factor TFIID subunit 6	TAFII70, TAFII80	P49848	ID, dysmorphic facial features, eye abnormalities, hypotonia, delayed psychomotor development, behavioral problems	I108T, I71T, R46C	(394, 395)
ARID1A (614607)	AT-rich interactive domain-containing protein 1A	BAF250A	O14497	ID, dysmorphic facial features, ear abnormalities, eye abnormalities, delayed psychomotor development, speech delay, hypotonia, seizures, brain abnormalities	Frameshift, termination	(355, 396, 397)
KMT2A (605130)	Histone-lysine N-methyltransferase 2A	ALL1, MLL1, HTRX	Q03164	ID, dysmorphic facial features, ear abnormalities, eye abnormalities, clinodactyly, hypotonia, delayed psychomotor development, speech delay, behavioral problems	Frameshift, termination	(398, 399)
ZC3H14 (617125)	Zinc finger CCCH domain-containing protein 14		Q6PJT7	ID	Termination	(306)
**KDM3B (****618846****)**	**Lysine-specific demethylase 3B**	**JMJD1B**	**Q7LBC6**	**ID, dysmorphic facial features, ear abnormalities, eye abnormalities, motor delay, speech delay, hypotonia, behavioral problems**	**D336G, E1731K, D1032V, termination**	(119, 120)
**CIC (****617600****)**	**Protein capicua homolog**	**Capicua homolog**	**Q96RK0**	**ID, delayed psychomotor development, developmental regression, seizures, brain abnormalities, hypotonia, behavioral problems**	**Termination, frameshift**	(400, 401)
POGZ (616364)	Pogo transposable element with ZNF domain		Q7Z3K3	ID, dysmorphic facial features, ear abnormalities, eye abnormalities, hypotonia, delayed psychomotor development, behavioral problems	Frameshift, termination, splice site	(215, 402, 403, 404, 405)
**ANKRD17 (****619504****)**	**Ankryin repeat domain-containing protein 17**	**GTAR**	**O75179**	**ID, developmental delay, dysmorphic facial features, eye abnormalities, speech delay, motor delay, seizures**	**Termination, G278V, A377T, L519P, R2434G, S1880P**	(406)
BPTF (617755)	Nucleosome-remodeling factor subunit BPTF	FAC1, Fetal Alzheimer Antigen	Q12830	ID, dysmorphic facial features, eye abnormalities, clinodactyly, hypotonia, delayed psychomotor development, seizures, brain abnormalities	Frameshift, A1924T, M2853R, termination, splicing, in-frame deletion	(407, 408)
**SPEN (****619312****)**	**Msx2-interacting protein**	**MINT, SHARP**	**Q96T58**	**ID, developmental delay, dysmorphic facial features, ear abnormalities, eye abnormalities, hypotonia, brain abnormalities, behavioral problems**	**Termination, frameshift**	(409)
NUP62 (271930)	Nuclear pore glycoprotein p62		P37198	ID, developmental arrest and regression, eye abnormalities, brain abnormalities	Q319P	(410)
HCFC1 (309541)	Host Cell Factor 1	HCF1	P51610	ID, delayed psychomotor development, seizures, hypotonia	A864T, G876S, A897V	(286, 287, 288, 289)

As shown in Figure 2, listed are OGT interactors catalogued by the Online Mendelian Inheritance in Man (OMIM) database as having an ID phenotype. Identification information for each protein included here are the Gene ID (with associated MIM identification), UniProt accession number, and alternative names by which the protein may be listed. *Highlighted Features of Neurodevelopmental Disorder* includes phenotypes seen in patients with causal mutations in that gene in relation to those features common to OGT-CDG patients. Example variants listed are variants in a particular interactor which have been found causal for neurodevelopmental disorders. Applicable references are listed. Proteins in **bold** have been linked to a neurodevelopmental disorder since the publication of the OGT interactome by Stephen *et al.* 2020 (60). Abbreviations used in this table include: ID (intellectual disability), ASD (autism spectrum disorder), and ADHD (attention-deficit/hyperactivity disorder).

OGT-CDG is an X-linked recessive disorder, and therefore it primarily affects males. In instances where OGT-CDG is inherited, the maternal parent tends to be unaffected by the disorder. X-inactivation, where one chromosome is preferentially inactivated, is often seen in disorders with X-linked intellectual disability (36). This skewing in females toward one X chromosome and the preferential inactivation of the other can be advantageous for survival particularly when there is a genetic defect on the inactivated X chromosome, as it will prevent the expression of the disease in that individual. Male progeny, however, do not share this protective effect and will show disease signs through the inheritance of the disease allele. This is the case with OGT-CDG, where unaffected females transmit the disorder to their sons, but the unaffected females are heavily skewed away from the disease allele, as opposed to the normal 50:50 inactivation of the *OGT* gene (37). Skewing of the X-chromosome in the maternal parent contributed to the prediction of causality of the OGT^L254F^ mutation in the family analysis (31). Interestingly, OGT^N567K^ represents a unique case where monozygotic female twins share a heterozygous missense mutation in *OGT* (34). A 98:2 skew in X inactivation was detected in both twins, while the maternal parent was unaffected. However, which X chromosome is inactivated is unclear. Despite sharing the same variant and showing the same skewing in X-inactivation, the severity of the OGT-CDG phenotype varied between the twins suggesting X-inactivation or neonatal environmental factors have a role in the severity of the phenotype in patients. Skewing also suggests that the wildtype allele must have a selective advantage in some fundamental process of early development.

In the clinical setting, there is a need to determine diagnostic techniques for addressing causality for ID. Initial key phenotypic characteristics to observe include cognitive disability and developmental delay, with eye abnormalities and behavioral abnormalities being additional strong indicators of an ID phenotype that could include an OGT-CDG diagnosis. Because of the variability of the dysmorphic features seen, pinpointing specific characteristics is difficult, other than generalizing a dysmorphic feature phenotype for OGT-CDG. However, these phenotypic diagnoses are not sufficient to confirm OGT-CDG. Exome sequencing should be used to identify what particular additional mutations a patient’s genome may harbor, and an exome database allows for ruling out common polymorphisms from unaffected individuals (9). Should a mutation in *OGT* appear, X-linked skewing of the maternal parent may provide evidence to the causal nature of the variant, particularly if the mother is unaffected with skewing towards the non-affected allele. Finally, patient cells can be assessed for changes relative to healthy controls. Previous work with OGT^L254F^, OGT^R284P^, and OGT^Δ155-177^ indicate OGT and OGA levels decrease in patient cells compared to wildtype controls, but no change in global O-GlcNAc levels is observed (30, 31). In OGT^L254F^ lymphoblastoids, *OGA* mRNA and *OGA* promoter-reporter expression both decrease, which provides a potential mechanism by which normal global O-GlcNAcylation is maintained in these cells. Through RNA-sequencing analysis, OGT^L254F^ lymphoblastoids are found to have a subset of disease-relevant differentially regulated genes compared to wild-type controls (31). More research is necessary to identify which genes may be optimal candidates and if this effect is seen for other variants, but this suggests that utilizing RNA-sequencing to search for specific genetic biomarkers may help in assigning causality of OGT-CDG variants.

## OGT-CDG variants exhibit variable altered biochemical properties

While the biochemical properties of the OGT-CDG variants studied to date are quite variable (Table 2), there are methods to predict pathogenicity. *In silico* analyses algorithms, such as Polyphen and SIFT (38, 39), have been used to predict whether OGT-CDG variants are likely deleterious (Table 2) (30, 31, 32). The conservation of amino acid residues can also be used to predict pathogenicity, as the pathogenic variants described to date affect residues that are conserved across vertebrate and invertebrate species (30, 31, 32, 33, 34, 35). Moreover, computational modeling of variants allows for predictions to be made as to the type of effect a variant might have on the enzyme (31, 32, 33, 34, 35). For example, TPR domain variants were predicted to result in steric clashes or loss of secondary structures while catalytic domain variants were predicted to affect substrate binding. Structural studies of OGT-CDG variants have allowed for these predicted changes to be examined. The crystal structures of OGT^N567K^ and OGT^N648Y^ suggest changes in the catalytic domain alter substrate binding (33, 34). Moreover, the crystal structure of OGT^L254F^ shows a small change in TPR 7, and this deviation propagates toward the N terminus showing that it destabilizes the interface between TPRs 6 and 7 (40). In further confirmation of the effects of TPR domain variants on the structure, the spring constants, which measure the elasticity of the TPR domain, were determined for OGT^L254F^, OGT^R284P^, and OGT^A319T^, and all three have alterations that could lead to changes in the ability of these enzymes to adapt to different substrates and complexes (41). Finally, thermal stability assays have also been employed to determine changes in protein stability with varying degrees of change for the TPR domain variants (Table 2) (30, 32, 40). Overall, studies examining the structure of OGT-CDG variants point toward domain-specific changes: TPR domain variants have altered structure which could affect protein-protein interactions, substrate selection, and stability, while catalytic domain variants have changes in the binding site of substrates.

**Table 2:** Summary table of OGT-CDG variant characterization

Variant	L254F	A259T	R284P	A319T	E339G
Domain	TPR	TPR	TPR	TPR	TPR
Polyphen Score	0.999	1.000	0.967	0.999	1.000
*In vitro* Glycosyltransferase Activity	• Normal activity (CKIIα) • ↓ activity (de-O-GlcNAcylated HEK293 cell lysate) • Normal activity (HEK293 overexpression system)	Normal activity (CKIIα)	• Normal activity (CKIIα) • ↓ activity (de-O-GlcNAcylated HEK293 cell lysate)	Normal activity (CKIIα)	Normal activity (CKIIα)
*In vitro* HCFC1 Glycosyltransferase Activity	Normal activity	Normal activity	Reduced activity	Normal activity	Normal activity
*In vitro* HCFC1 Protease Activity	Normal activity	Normal activity	Reduced activity	Normal activity	Normal activity
Kinetic Analysis	• *K*_*m*_ 1.86-fold change, *k*_*cat*_ 0.78-fold change *versus* WT (CKIIα protein substrate) • K_m_ and *V*_*max*_ unchanged (peptide substrate)	*K*_*m*_ 1.29-fold change, *k*_*cat*_ 0.78-fold change *versus* WT (CKIIα protein substrate)	*K*_*m*_ 1.29-fold change, *k*_*cat*_ 0.78-fold change *versus* WT (CKIIα protein substrate)	*K*_*m*_ 2.07-fold change, *k*_*cat*_ 0.73-fold change *versus* WT (CKIIα protein substrate)	*K*_*m*_ 1.71-fold change, *k*_*cat*_ 0.89-fold change *versus* WT (CKIIα protein substrate)
Dimerization	Normal dimerization of recombinant TPR domain	Normal dimerization of recombinant TPR domain	Normal dimerization of recombinant TPR domain	Normal dimerization of recombinant TPR domain	Normal dimerization of recombinant TPR domain
Thermal Stability	↓	↓	↓	↓	↓
Global O-GlcNAc Levels	Unchanged (hESCs)	Unchanged (hESCs)	Unchanged (hESCs)	N/A	Unchanged (hESCs)
Global OGT Levels	Unchanged (hESCs)	Unchanged (hESCs)	Unchanged (hESCs)	N/A	Unchanged (hESCs)
Global OGA Levels	Unchanged (hESCs)	Unchanged (hESCs)	Unchanged (hESCs)	N/A	Unchanged (hESCs)
Gene Expression	• Altered (patient derived lymphoblastoids) • Altered (hESCs)	Altered (hESCs)	Altered (hESCs)	N/A	Altered (hESCs)
Pluripotency Markers	Unchanged *POU5F1* (Oct4), *SOX2*, *NANOG* (hESCs)	Unchanged *POU5F1* (Oct4), *SOX2*, *NANOG* (hESCs)	Unchanged *POU5F1* (Oct4), *SOX2*, *NANOG* (hESCs)	N/A	Unchanged *POU5F1* (Oct4), *SOX2*, *NANOG* (hESCs)
Animal and Cell Models	*Drosophila*: • Non-performers in habituation learning • ↑ synaptic bouton number, ↑ NMJ length (not significantly), ↑ number of NMJ branches	N/A	*Drosophila*: • ↓ climbing speed • Non-performers in habituation learning (homozygous) • Deficits in habituation learning (heterozygous) • ↑ synaptic bouton number, ↑ length of NJM, ↑ perimeter of NMJ, ↑ number of NMJ branches	*Drosophila*: • Deficits in habituation learning (homozygous) • ↑ synaptic bouton number, ↑ NMJ length (not significantly), ↑ NMJ length	N/A
References	(31, 32, 40, 49)	(32)	(30, 32, 49)	(32, 49)	(32)


Variant	Δ155–177	N567K	N648Y	C921Y
Domain	TPR	Catalytic	Catalytic	Catalytic
Polyphen Score	N/A	0.998	1.000	1.000
*In vitro* Glycosyltransferase Activity	N/A	↓ (TAB1)	↓ (TAB1)	↓ (TAB1)
*In vitro* HCFC1 Glycosyltransferase Activity	N/A	↓ activity	N/A	↓ activity
*In vitro* HCFC1 Protease Activity	N/A	↓ activity	N/A	Normal activity
Kinetic Analysis	N/A	*K*_*m*_ 1.45-fold change, *k*_*cat*_ 0.17-fold change, *k*_*cat*_*/K*_*m*_ 0.08-fold change *versus* WT (peptide substrate)	*K*_*d*_ 6.88-fold change *versus* WT (fluorescent probe)	*V*_*max*_ 0.25-fold change, *K*_*m*_ 0.18-fold change, *k*_*cat*_*/K*_*m*_ unchanged *versus* WT (peptide substrate)
Dimerization	N/A	N/A	N/A	N/A
Thermal Stability	↓ thermal stability	N/A	Normal thermal stability	Normal thermal stability
Global O-GlcNAc Levels	• Unchanged (patient derived fibroblasts) • Unchanged (iPSCs) • ↓ (differentiated iPSCs) • ↓ (NPCs)	Unchanged (pluripotent mESCs)	↓ (mESCs)	• ↓ (undifferentiated, mESCs) Unchanged (mESCs, clonogenic conditions)
Global OGT Levels	• ↓ (patient derived fibroblasts) • ↓ (iPSCs) • ↓ (differentiated iPSCs) • ↓ (NPCs)	Unchanged (pluripotent mESCs)	Unchanged (mESCs)	• ↑ (undifferentiated mESCs) ↑ (mESCs, clonogenic conditions)
Global OGA Levels	• ↓ (patient derived fibroblasts) • ↓ (iPSCs) • ↓ (differentiated iPSCs) • ↓ (NPCs)	• ↓ (pluripotent mESCs) • Unchanged (differentiated mESCs)	↓ (mESCs)	• Unchanged (undifferentiated mESCs) Unchanged (mESCs, clonogenic conditions)
Gene Expression	• Unchanged *OGT*, *OGA* mRNA (patient derived fibroblasts) • ↑ *OGT*, ↓ *OGA* mRNA (iPSCs) • Unchanged *OGT*, ↓ *OGA* mRNA (differentiated iPSCs) • ↑ *NEFH*, all other ectoderm markers unchanged (differentiated iPSCs) • Unchanged *OGT*, *OGA* mRNA (NPCs)	↑ GABPA mRNA (mESCs)	N/A	Unchanged *Ogt*, ↓ *Oga* mRNA (undifferentiated mESCs)
Pluripotency Markers	• Unchanged *POU5F1* (Oct4), *SOX2*, *NANOG* mRNA, Unchanged NANOG, OCT4, SOX2 protein (iPSCs)	Unchanged *Sox2, Oct4* during first 6 days of differentiation (mESCs)	N/A	• Unchanged *Oct4* and *Sox2* mRNA, Oct4 and Sox2 protein (undifferentiated mESCs) ↓ Oct4, Sox2 protein (mESCs, clonogenic conditions)
Animal and Cell Models	• Unchanged pluripotency, differentiation, and morphology (iPSCs) • Unchanged neural progenitor markers (NPCs) • Fewer and larger rosettes, larger apical lumens (NPCs)	*Drosophila*: • ↓ O-GlcNAc (adult heads) mESCs: • ↓ neurite length • ↓ proteolytic cleavage of HCFC1 Mice: • Altered inheritance distribution • ↓ Global GlcNAc, ↓ OGA protein, ↓ OGT protein (brain) • ↓ *Oga*, ↓ *Ogt* mRNA, ↓ DIs, ↓ PEs (brain) • ↓ body weight, ↓ nose-to-tail length, ↑ pancreas weight, slim appearance • ↓ brain weight	Mice: • ↓ Global GlcNAc, ↓ OGA protein, ↓ OGT protein (brain) • ↓ *Oga*, ↓ *Ogt* mRNA, ↓ DIs, ↓ PEs (brain) • ↓ body weight, ↓ nose-to-tail length, ↑ pancreas weight, slim appearance • ↓ brain weight	*Drosophila*: • ↓ O-GlcNAc, ↑ OGT protein (adult heads) • ↓ O-GlcNAc, ↑ OGT protein (embryos) • ↓ O-GlcNAc, unchanged OGT protein (larvae) • ↑ ectopic bristle penetrance • ↓ NMJ area, ↓ NJM length, ↓ NMJ bouton numbers • ↓ total sleep, ↓ duration and more frequent sleep bouts mESCs: ↓ alkaline phosphatase staining (clonogenic conditions) Mice: • ↓ Global GlcNAc, ↓ OGA protein, ↓ OGT protein (brain) • ↓ *Oga*, ↓ *Ogt* mRNA, ↓ DIs, ↓ PEs (brain) • ↓ body weight, ↓ nose-to-tail length, ↓ body fat mass, ↑ lean body mass, ↓ glycemia, ↑ pancreas weight, slim appearance • ↓ skull length, ↓ skull size, ↓ absolute brain weight, rounder and smaller skull Hypothesized anxiety phenotype, compulsive behavior, altered spatial working memory
References	(30, 44)	(34, 50)	(33, 50)	(35, 50, 51)

Summarized are characteristics of the six Tetratricopeptide repeat (TPR) domain variants and three catalytic domain variants of OGT discussed in this review. This table shows the results of characterizations of variants *in vitro,* *in cellulo,* and *in vivo* alongside phenotypes observed *in vivo*. Abbreviations used within include WT (wild-type), TPR (Tetratricopeptide repeat), CKIIα (casein kinase II alpha), TAB1 (TAK-1 binding protein), hESCs (human embryonic stem cells), iPSCs (induced pluripotent stem cells), mESCs (mouse embryonic stem cells), NPCs (neural progenitor cells), NMJ (neuromuscular junction), DI (detained introns), PE (decoy exon), ↑ (upregulated or increased), ↓ (downregulated or decreased), N/A (information unavailable or not determined).

Unlike structural effects, OGT-CDG variants have divergent biochemical properties that do not appear to be domain-specific. All variants to date have been tested for their ability to glycosylate substrates and some for their ability to cleave HCFC1 (Table 2). OGT^L254F^ raises global O-GlcNAc levels in a HEK293 overexpression system to the same degree as wildtype (31), but this same variant has a reduced ability to modify de-O-GlcNAcylated HEK293 lysates, as was also seen for OGT^R284P^ (30, 40). However, all characterized TPR domain variants glycosylate known OGT substrate Casein Kinase II alpha (CKIIα), though with a modest increase in their *K*_M_ values for the protein substrate (31, 32, 42). This is in contrast to the catalytic domain variants which are greatly impaired in their ability to modify the protein substrate TAK-1 binding protein (TAB1) (33, 34, 35, 43). Kinetics analysis of catalytic domain variants also reveals markedly altered kinetics for peptide substrates (33, 34, 35). *In vitro* assays investigating the ability of OGT variants to cleave and glycosylate HCFC1 show an inability for OGT^N567K^ to cleave or glycosylate HCFC1, while when examining the TPR domain variants only OGT^R284P^ has a slight decrease in this enzymatic activity (32, 34). Despite the variability of OGT-CDG biochemical properties (Table 2), these results inform future studies. In the future characterization of OGT-CDG variants, HCFC1 glycosylation and cleavage should be probed as this defect may play a role in the mechanism of OGT-CDG for some variants. Furthermore, glycosylation assays show clear defects for catalytic domain variants while TPR domain variants have modest changes in kinetics for a protein substrate. While TPR domain variants have modest changes in glycosylation activity, a reduction in glycosyltransferase activity nonetheless suggests this group of genetic disorders is in fact a disorder of glycosylation. However, the TPR domain variants likely only have a subset of substrates that are differentially O-GlcNAcylated compared to the drastic O-GlcNAcylation defects seen for catalytic domain variants. While these studies provide useful information about the OGT-CDG enzymes, model systems, including relevant cell types and animal models, will help elucidate the intricacies underlying the OGT-CDG phenotype.

## Model organisms provide opportunities to elucidate the OGT-CDG mechanisms

Initial experimental work on cell and animal models of OGT-CDG has focused on identifying key phenotypic consequences of disrupting OGT function, which may indicate processes particularly sensitive to OGT dysfunction. OGT is known to be essential for mammalian embryogenesis (27) and many phenotypes seen in OGT-CDG patients indicate defects in early development (*i.e.* developmental delay, Table 1). Therefore, studies have focused on relevant cell types, namely human and mouse embryonic stem cells (ESCs) (32, 33, 34, 35) and more recently patient cells reprogrammed into induced pluripotent stem cells (iPSCs) (44). Catalytic domain variants have been modeled in mouse ESCs (mESCs), and these models demonstrate heterogeneous alterations in global O-GlcNAcylation, OGT, and OGA levels (Table 2) (33, 34, 35). OGT^C921Y^ and OGT^N648Y^ variants result in lower levels of global O-GlcNAc in mESCs. The OGT^N567K^ variant does not show a decrease in global O-GlcNAc, however, this may be explained by decreased OGA levels in cells modeling this mutation, a phenotype also observed in OGT^N648Y^ mESCs. Furthermore, OGT^C921Y^ shows an increase in OGT levels. Thus, while the changes in the O-GlcNAc cycling enzymes and global O-GlcNAc levels are different among the catalytic domain variants, the results suggest that the cells are attempting to maintain O-GlcNAc homeostasis in response to the variant enzymes. Further heterogeneity of the catalytic domain variants is seen in their ability to process HCFC1. While OGT^C921Y^ is deficient in its ability to glycosylate HCFC1 *in vitro*, there is no apparent effect on HCFC1 cleavage *in vitro* or in mESCs (35). This is in contrast to OGT^N567K^, which shows a marked decrease in the proteolytic fraction of HCFC1 (34). Moreover, OGT^N567K^ mESCs shows a change in expression of a gene regulated by HCFC1, Ets transcription factor GA-binding protein subunit alpha (GABPA) (34). Given that HCFC1 proteolysis is required for normal function in gene expression (13, 14, 15, 16), it is possible that HCFC1 plays a role in the OGT-CDG phenotype for some variants. Furthermore, iPSCs have been generated for a mutation resulting in the deletion of exon 4 of the TPR domain, OGT^Δ155-177^ (44). While global O-GlcNAc levels are maintained during an undifferentiated state, upon differentiation towards an ectodermal fate global O-GlcNAc levels are altered for the variant in comparison to a corrected iPSC control. OGT^Δ155-177^ results in a decrease of OGT levels but maintains O-GlcNAc homeostasis by downregulating OGA levels. It appears the homeostatic mechanism regulating the expression of OGT and OGA is insufficient to maintain normal O-GlcNAcylation despite a continued decrease in OGA levels in neural progenitors. This heterogeneity in the homeostasis of O-GlcNAc and its cycling enzymes is not seen when TPR domain variants are modeled in human ESCs (hESCs). Four TPR domain variants (OGT^L254F^, OGT^A259T^, OGT^R284P^, and OGT^E339G^) show similar levels of global O-GlcNAc, OGT, and OGA (32). The divergence in the models suggests that there may be multiple underlying mechanisms to the OGT-CDG phenotype or differences arising from the cell types used. Some degree of variability seen in patients may arise not only due to differences in genetic background but also due to heterogeneous effects of *OGT* mutations on the multiple functions fulfilled by OGT.

Beyond alterations to global O-GlcNAc homeostasis, studies have also attempted to uncover downstream effects of OGT-CDG variants on cell and animal models. Some variants have been probed for changes in the pluripotency network, namely SOX2 and OCT4 (Table 2). TPR domain variants modeled in hESCs showed no differences in the expression of SOX2 or OCT4 (32). However, OGT^C921Y^ shows decreased levels of SOX2 and OCT4 protein levels (35), while OGT^N567K^ mESCs and iPSCs of OGT^Δ155-177^ have normal levels of SOX2 and OCT4 (34, 44). The changes in SOX2 and OCT4 protein levels could explain the altered ability of OGT^C921Y^ mESCs to maintain pluripotency upon withdraw of Leukemia Inhibitory Factor, LIF. Interestingly, upon differentiation of mESCs, OGT^N567K^ shows a decrease in neurite length, while OGT^Δ155-177^ iPSCs show fewer and larger neural rosettes (34, 44). Alterations in neural rosette formation *in vitro* further point toward early developmental changes as neural rosette formation is reminiscent of secondary neurulation *in vivo* (45). However, an important caveat to comparing results from human and mouse cell models is that transcriptional networks maintaining an undifferentiated state differ substantially between these two organisms (46). While variable, these results from models in mESCs and iPSCs provide evidence for multiple downstream targets being affected. Additionally, the transcriptomic profiling of four different TPR domain variants (OGT^L254F^, OGT^A259T^, OGT^R284P^, and OGT^E339G^) in hESCs shows a consistent phenotype. In this model, several pathways are found to be affected across the four mutations assessed. Notably, genes belonging to “mesoderm development” and “ectoderm development” gene ontology (GO) terms are significantly dysregulated (32). Altered expression of ectoderm and mesoderm genes could explain the ID as well as dysmorphic facial features and clinodactyly often seen in patients (Table 1). An additional pathway of interest downregulated across the four TPR domain variants is the Liver X Receptor/Retinoid X Receptor (LXR/RXR) activation pathway (32). This pathway has been implicated in dopaminergic neuron development, which may be consequential for memory and learning deficits seen in patients (47, 48). Overall, cell models to date have elucidated that gene expression is dysregulated and that the effects of OGT-CDG variants are cell-type specific. Beyond alterations in *OGT* and *OGA* mRNA expression, four TPR domain variants have altered gene expression in hESCs, and OGT^N567K^ has dysregulated expression of GABPA in mESCs pointing toward a common downstream impact on gene expression for both TPR domain and catalytic domain variants. Furthermore, the study utilizing iPSCs shows that the cell type is essential for uncovering the effects of OGT-CDG variants and further points toward the neural lineage as sensitive to perturbations in OGT function.

While disease-relevant cell models are invaluable for understanding the role of OGT in the earliest stages of development, many features of the OGT-CDG phenotype (Table 1) can only be recapitulated in animal models. OGT-CDG variants have been modeled in both *Drosophila* and mice, though only catalytic domain variants have been modeled in the latter (Table 2) (49, 50, 51). Broadly, all catalytic domain mutations modeled in either *Drosophila* (equivalent to OGT^N567K^ and OGT^C921Y^) or mice (equivalent to OGT^N567K^, OGT^N648Y^, and OGT^C921Y^)reduce global O-GlcNAcylation in brain tissue to varying degrees, while TPR domain mutations modeled in *Drosophila* (equivalent to OGT^L254F^, OGT^R284P^, and OGT^A319T^) do not broadly alter O-GlcNAc levels. In mice and consistent with mESC models, the three catalytic domain variants result in decreased OGA protein levels, at least in part through decreased mRNA levels (50). Unexpectedly, OGT protein levels are also significantly decreased in mouse models of OGT-CDG. This decrease in OGT protein levels likely occurs through posttranscriptional or posttranslational mechanisms, as OGT mRNA levels are significantly increased in these mice, unlike in cell models of OGT-CDG (50). These findings further underscore the need to better understand the mechanisms governing O-GlcNAc cycling, including genetic regulation of OGT and OGA, in different cell types and various stages of development and how these are affected by OGT-CDG mutations.

Beyond changes in O-GlcNAc homeostasis, mice and *Drosophila* models provide opportunities to study anatomical and behavioral effects caused by the OGT-CDG variants (Table 2). Behavioral assays in *Drosophila* suggest TPR domain variants (equivalent to OGT^R284P^ and OGT^A319T^) disrupt memory (49). In particular, habituation in these flies is disrupted, which can be rescued by knocking out OGA or by mutagenizing a key catalytic residue. Similarly, knocking out OGA can rescue sleep defects seen in a *Drosophila* model of catalytic domain variant OGT^C921Y^ (51). These results suggest that behavioral defects in *Drosophila* models of OGT-CDG may be conveyed through reduced O-GlcNAcylation. Furthermore, the effects of OGT-CDG variants on synaptic development have been assayed through imaging morphological features of the *Drosophila* larval neuromuscular junction (NMJ), which is the synaptic connection between neurons and muscle cells (49). TPR and catalytic domain variants display inconsistent results regarding the development of this synapse (49, 51). TPR domain mutations increase growth at this axonal terminal, resulting in the formation of more NMJ boutons, which are recognizable structures that contain synaptic vesicles (49). Conversely, the OGT^C921Y^ variant modeled in the fly results in decreased growth at the NMJ, characterized by reduced bouton number and NMJ area. This is phenocopied in larvae homozygous for a catalytically dead variant of OGT and partially rescued by restoring O-GlcNAcylation through knocking out OGA, suggesting this phenotype may be conveyed through impaired O-GlcNAc cycling. In mouse models of catalytic domain variants, low birth weight, microcephaly, and dysmorphic skull features seem to recapitulate the developmental delay, microcephaly, and dysmorphic features seen in patients (Tables 1 and 2) (50). Furthermore, mice with the OGT^C921Y^ variant show impairments in spatial memory, increased anxiety, and hyperactivity, though hyperactivity is not recapitulated in *Drosophila* (50, 51). Because OGT-CDG patient phenotypes can be reproduced in animal models, including the symptom heterogeneity observed, these models can be used for further study of downstream targets of OGT that may contribute to the mechanisms by which the disorder is conveyed.

## The neuroectodermal lineage is sensitive to perturbations in O-GlcNAc homeostasis

The neural-specific phenotype in OGT-CDG patients and the variable results from animal and cell models suggest that there are particular cell types that might be susceptible to alterations in OGT function and O-GlcNAc cycling, especially during early development. In a zebrafish model, *ogt* transcripts are high throughout the developing embryo pre-gastrulation but have a more localized expression in the brain post-gastrulation (52). In humans, alterations in O-GlcNAc levels *in utero* caused by gestational diabetes link OGT to neural tube defects (53, 54). In mammals, it has been shown that global O-GlcNAc levels decrease over-development (55). Moreover, multiple studies indicate O-GlcNAc homeostasis is required for normal differentiation of neuroectodermal lineage cells. Perturbations in O-GlcNAc cycling enzymes, and thus altered global O-GlcNAc levels, result in altered neuronal differentiation (56, 57, 58, 59). Furthermore, these alterations in differentiation are accompanied by changes in gene expression of neurogenic transcription factors as well as synaptic proteins. A decrease in global UDP-GlcNAc levels observed during differentiation of embryonic cells into neural lineage cells (56) could serve as a metabolic reprogramming mechanism by which OGT-mediated expression of genes is down-regulated during differentiation (Fig. 1*B*). The most recent study utilizing iPSCs generated from a patient further confirms the sensitivity of the ectodermal lineage to changes in O-GlcNAc homeostasis (44). Thus, O-GlcNAc homeostasis during development may cause certain cell lineages, such as the neuroectodermal lineages, to be sensitive to alterations in O-GlcNAc cycling and may lead to the OGT-CDG phenotype seen in patients. Furthermore, common alterations in glycosyltransferase activity and changes in gene expression, seen most notably in the TPR domain variant hESCs and in patient lymphoblastoids of OGT^L254F^, suggest that in part the mechanism behind OGT-CDG lies in hypoglycosylation of key substrates leading to altered gene expression and neurodevelopment. In an attempt to determine the most relevant possible mechanistic targets for OGT-CDG studies, we chose to evaluate a previous interactome generated for OGT’s TPR domain in HeLa cells (60). In the next section, we will discuss how the OGT interactome is enriched for proteins that are causal for an ID phenotype and regulate gene expression at multiple levels.

## The OGT interactome is enriched for causal intellectual disability proteins

With the variable biochemical and phenotypic features of causal OGT variants, the next step in understanding the mechanisms underlying the phenotype is to determine relevant targets that could be conveying the phenotype. Since the phenotype seen in patients includes ID and developmental delay regardless of whether the variant lies in the TPR or catalytic domain, there are likely downstream targets of OGT that are hypoglycosylated and are responsible for normal neurodevelopment. This hypoglycosylation is likely a result of reduced catalytic activity from variants of the catalytic domain or as a result of changes in protein-protein interactions resulting from the altered structure of the TPR domain from variants of the TPR domain. Given the key role of OGT in transcriptional regulation and the changes in gene expression seen for the OGT-CDG variants tested, downstream targets are likely responsible for proper spatiotemporal gene expression during embryonic development and downstream targets could be required for normal neuronal functioning.

**Figure 3:**
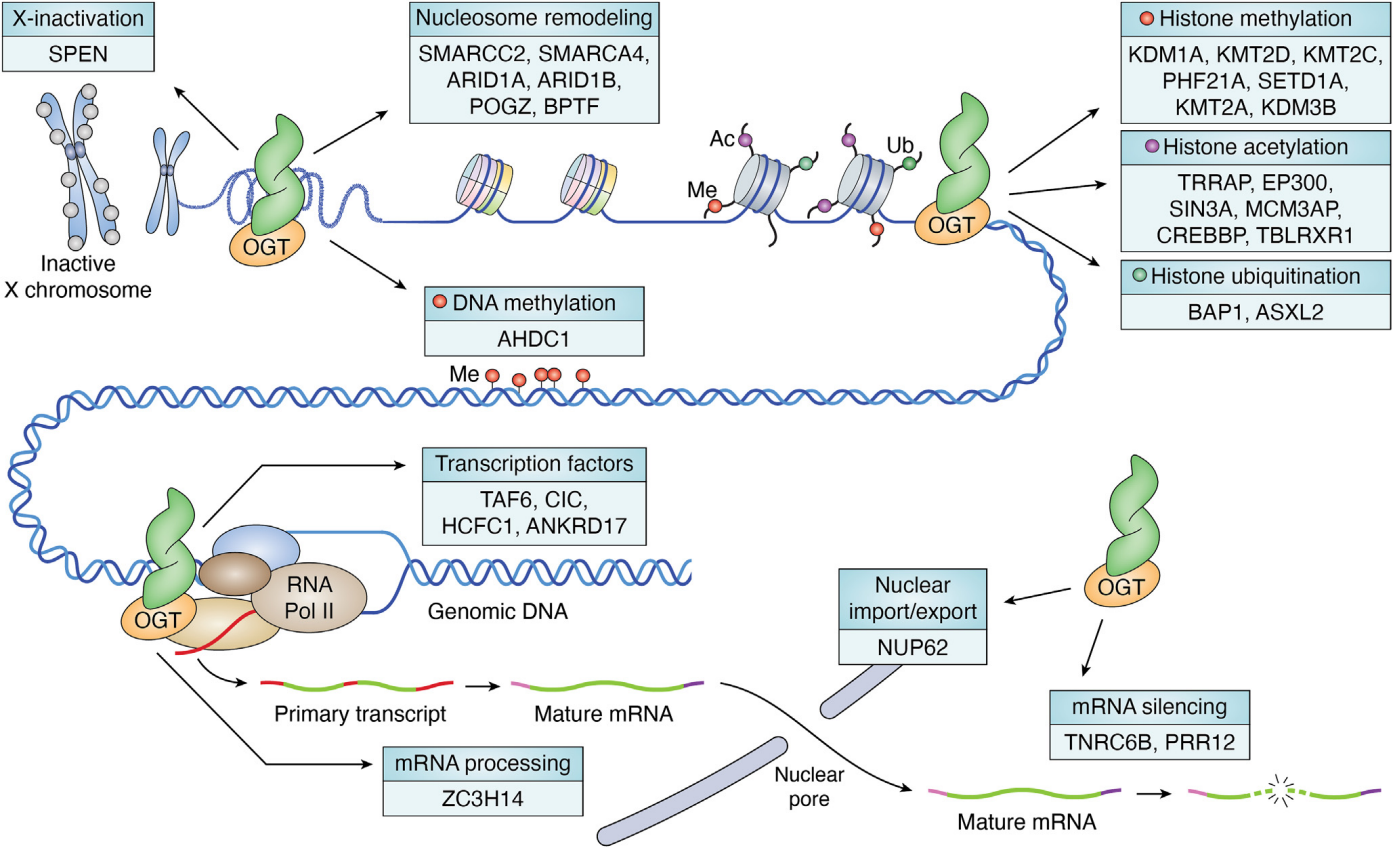
**OGT interactors involve multiple aspects of transcription regulation.** OGT interactors involve multiple aspects of the regulation of gene expression. All OGT interactors are not only predicted to be causal for disorders with ID phenotypes, like seen in OGT-CDG, but they also all have roles in the regulation of gene expression. Depicted here is each of the 32 interactors discussed in this review and where each plays a role in regulating gene expression: X-inactivation, nucleosome remodeling, histone methylation, histone acetylation, histone ubiquitination, DNA methylation, transcription factors, mRNA processing, nuclear import/export, and mRNA silencing. Ac, acetylation; Me, methylation; RNA Pol II, RNA polymerase II; Ub, ubiquitination.

Previously, a bioinformatics approach using sequence conservation, structural data, and clinical data was used to determine possible targets of OGT that are also linked to neurodevelopmental disorders (61). Many targets with mapped O-GlcNAc sites were identified as potential conveyors of the ID phenotype, and these substrates represent targets worth investigating in the context of OGT-CDG. Here, we expand on this list by discussing interactors of OGT identified in a proximity labeling (BioID) approach for OGT’s TPR domain (60). This basal OGT interactome in HeLa cells contained 24 proteins with OMIM-catalogued neurodevelopmental disorders at the time of initial publication out of 115 total (Table 1). Some of these interactors were also identified using the previously mentioned bioinformatics approach including ARID1A, EP300, and HCFC1(61). This list of ID-linked interactors has since expanded to 32 out of a total of 115, and these data suggest that interactors and substrates of OGT are enriched for proteins implicated in neurodevelopment (Table 1, **bold**) as has been shown for the *Drosophila* O-GlcNAcome (49). These interactors represent a diverse array of proteins, but broadly all are regulators of gene expression (Fig. 3). This is in line with other generated OGT interactomes, in which regulators of transcription are enriched (62, 63, 64, 65). Furthermore, the interactors discussed herein are represented in the other interactomes from other cell types as well as from mice. While not all of these interactors have mapped O-GlcNAc sites (Fig. 2), many of these have mapped O-GlcNAc sites in mESCs (*i.e.* SIN3A, HCFC1, KMT2D, ZC3H14, POGZ, PHF21A, ANKRD17, SPEN, and TAF6) (66). Additionally, SIN3A, HCFC1, KMT2D, ZC3H14, and POGZ all had differentially enriched O-GlcNAc sites upon differentiation with Retinoic Acid (RA) towards an ectodermal fate. While this list of interactors does not represent an exhaustive list of potential targets, they are enriched for neurodevelopmental disorders, represent key regulators of gene expression, and represent both substrates and interactors of OGT which uniquely positions them as critical targets of investigation to understand potential mechanisms of OGT-CDG as well as to better understand the complex role of OGT in regulating gene expression.

**Figure 4:**
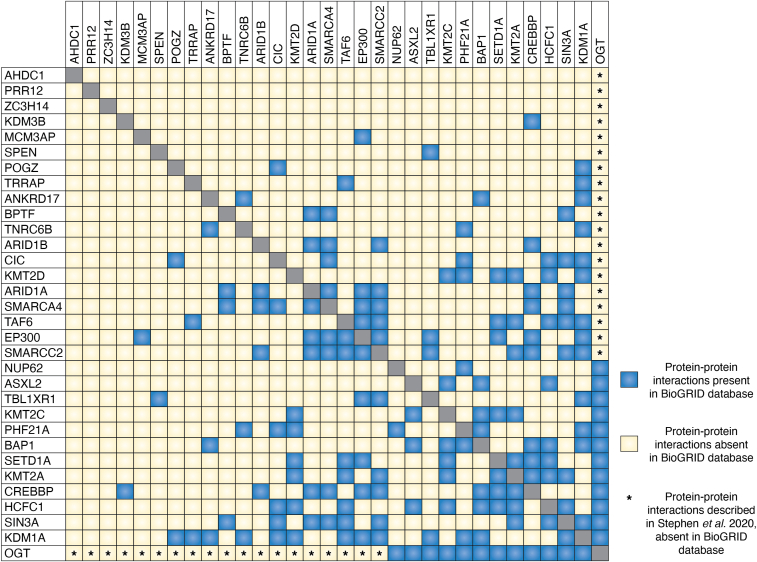
**Direct interactions between identified OGT interactors**. Using the Biological General Repository for Interaction Datasets (BioGRID) database, we searched which proteins in our group of OGT interactors (Table 1) interacted with other proteins within the subset. If BioGRID contained publications indicating an interaction between two proteins, a *blue box* is present (example: OGT-HCFC1). A lack of interaction between interactors in BioGRID is denoted by a *yellow box* (example: PRR12-AHDC1). A *yellow* box with an asterisk indicates an interaction detected between OGT and another protein that was identified by Stephen *et al.* 2020 (60) but not in the BioGrid database.

These interactors consist of many epigenetic regulators of histone modifications including histone methylation, acetylation, and ubiquitination, as well as regulators of mRNA processing, export, and silencing (Fig. 3). In the context of O-GlcNAc biology, some interactors have well-established links to OGT while others are uncharacterized in this regard. Some interactors, like HCFC1 and NUP62, have numerous mapped O-GlcNAc sites (Fig. 2), and only a few interactors, like ARID1B and BAP1, have only one or no mapped O-GlcNAc sites (Fig. 2). Furthermore, many of these proteins have known interactions with each other (Fig. 4) suggesting the existence of OGT-containing multiprotein complexes (Fig. 1*B*). In support of this concept, several established complexes were recovered in the OGT interactome including many members of the BAF (mSWI/SNF) complex and the Polycomb Repressive Deubiquitinase (PR-DUB) complex. The OGT interactome provides disease-relevant targets that should be investigated to understand possible OGT-CDG mechanisms as well as to better understand the role of OGT in the regulation of gene expression.

## Histone methylation

The OGT interactome is enriched for proteins involved in the regulation of histone methylation including four histone methyltransferases, two histone demethylases, and one reader/scaffolding protein that is all causal for neurodevelopmental disorders (Fig. 3) (60, 67, 68). KMT2A, KMT2C, KMT2D, and SETD1A (KMT2F) are all catalytic subunits of the MLL/SET1 complexes responsible for H3K4me1/2/3 histone methylation and transcriptional activation of genes (Fig. 3) (69, 70, 71, 72, 73, 74, 75, 76, 77, 78, 79). Numerous studies have linked dysfunction or insufficiency of these histone methyltransferases to alterations in neurodevelopment and neuronal function (80, 81, 82, 83, 84, 85, 86, 87, 88, 89, 90, 91, 92, 93, 94, 95). Moreover, many of the phenotypes observed in models of ID caused by mutations in these methyltransferases can be rescued both *in vitro* and *in vivo* (82, 85, 87, 93, 96, 97). In particular, pyruvate supplementation and inhibition of RAS/MAPK, HDACs, and KDM1A have been shown to rescue some or all of the observed phenotypes. Glucosamine supplementation and OGA inhibition have been previously suggested as an intervention for OGT-CDG patients (98), though these supplementations have not been tested *in vitro* for OGT-CDG variants. KDM1A (LSD1) was also enriched in the OGT interactome, but no studies have mapped O-GlcNAc sites to the protein (Fig. 2). However, the histone methylation reader and scaffold for the CoREST complex (99, 100, 101), PHF21A, has mapped O-GlcNAc sites that could be functionally relevant. Both KDM1A and PHF21A have been linked to neurodevelopmental disorders and have been studied in functionally relevant models (102, 103, 104, 105, 106, 107, 108, 109, 110, 111, 112, 113, 114, 115, 116, 117, 118). Together with other members of the CoREST complex, CoREST, and HDAC1/2, KDM1A functionally silences genes that are normally expressed in neuronal cell lineages. Furthermore, KDM1A has a neuron-specific isoform that is catalytically inactive but is believed to be responsible for the de-repression of neuron-specific genes by inhibiting the function of the ubiquitous KDM1A isoform. Any role of OGT in the regulation of this complex would be worth studying in relevant neural cell lines. KDM3B, another histone demethylase, has recently been reported to be causal for a neurodevelopmental disorder (119, 120), and one study has shown a link between this histone demethylase and neurodevelopmental and neuronal function (121). Lastly, JMJD1C is another interactor identified that has functional roles in histone methylation, and two recent reports have linked it to a neurodevelopmental disorder, but the association needs further confirmation (122, 123). Functionally, perturbing O-GlcNAc levels during neurogenesis results in alterations of histone methylation, and in particular, the levels of H3K4me3 on neurogenic transcription factor promoters increase alongside an increase in KMT2D and a decrease in KDM1A *in vitro* (58). Moreover, a rat model of maternal hyperglycemia showed an increase in KMT2A levels. Furthermore, EZH2, the catalytic subunit of the Polycomb repressive complex 2 (PRC2) and ID causal protein (MIM#277590), showed changes in phosphorylation accompanied by a change in the repressive H3K27me3 histone modification. While this study used chemical inhibitors to raise O-GlcNAc levels, it is clear that perturbing the O-GlcNAc homeostasis during neurogenesis causes dysregulation of gene expression at least in part by changes in histone methylation (58). The role of OGT in histone methylation is a pertinent question not only for understanding its role in transcription regulation but also for understanding the mechanisms responsible for the OGT-CDG phenotype. When investigating causal OGT-CDG mutants, studies should consider the methylation of histones in relevant models given that OGT interacts with more than seven enzymes responsible for proper histone methylation during development.

## Histone acetylation

Further supporting the investigation of epigenetic modifications in the OGT-CDG phenotype is the enrichment of OGT interactors involved in histone acetylation. CBP, EP300, TRRAP, MCM3AP, TBLRXR1, and SIN3A all play a role in histone acetylation or deacetylation either enzymatically or as scaffolds for HDAC-containing complexes (Fig. 3). CBP and EP300 are both histone acetyltransferase as well as modifiers of other proteins (124, 125, 126, 127, 128, 129, 130), and both are causal for neurodevelopmental phenotypes with overlapping presentations (Table 1). Their links to neurodevelopment and neuronal function have been well studied (131, 132, 133, 134, 135, 136, 137, 138, 139, 140, 141), and a possible link to OGT-CDG has been discussed previously (61). Some studies have shown that the O-GlcNAc cycling enzymes cooperate with CBP/EP300 acetylation of substrates (124, 125, 142), suggesting coregulation of transcription between CBP, EP300, and OGT. Both TRRAP and MCM3AP function as scaffolds for histone acetyltransferase complexes such as SAGA, Tip60, and TREX-2 complexes (143, 144, 145, 146, 147, 148, 149, 150, 151, 152, 153, 154, 155, 156, 157, 158). Studies have shown that TRRAP is necessary for normal development including for neural lineage cells (143, 159, 160, 161, 162). However, the role of MCM3AP in neurodevelopment and the functioning of the adult nervous system is less well understood (163). OGT could play a role in stability in the complexes containing TRRAP and MCM3AP either through modification of these scaffolds or by non-catalytic functions (Fig. 1*A*), but further investigation is required to uncover if either of these is likely. SIN3A and TBLRXR1 both play a scaffolding role as well, but these proteins are involved in the deacetylation of histones and thus transcription repression (164, 165, 166, 167, 168, 169, 170, 171, 172, 173, 174). TBLRXR1 is a part of the NCOR/SMRT complex, which is responsible for gene repression of neuronal genes. TBL1XR1 is causal for a neurodevelopmental phenotype (175, 176), but recent reports also suggest that NCOR1, NCOR2 (SMRT), and HDAC3 are also responsible for an ID phenotype (177), providing a basis for investigation of the relevance of the complex and its members in the OGT-CDG phenotype. The last interactor in this section, SIN3A, has known roles in neuronal function and neurodevelopment (167, 178, 179, 180, 181, 182, 183) and has also been shown to partner with OGT *in vivo* to regulate gene expression (164, 184). Moreover, SIN3A and TBLRXR1 also tie in another predicted putative conveyor of the OGT-CDG phenotype, MECP2 (61). SIN3A and TBLRXR1 both have links to MECP2 function and Rett syndrome (RTT) *in vivo* (185, 186, 187, 188, 189). SIN3A and HDAC1/2 levels are altered along with histone acetylation in the RTT mouse cortex, and RTT causal mutations disrupt the binding between TBLRXR1 and MECP2. If OGT-CDG mutations in the TPR domain cause disruptions in normal protein-protein interactions, it is worth investigating how the phenotype could be conveyed through all of these proteins, especially in light of a recent report suggesting that RTT etiological site T203 is a functional O-GlcNAc site with disruptions in normal neuronal function and morphology *in vivo* (190). Future studies with the aim of site mapping O-GlcNAc sites would determine what sites might differ between wildtype OGT and the OGT-CDG variants in relevant model systems. The enrichment of OGT’s interactome for proteins involved in histone acetylation suggests examining the acetylation status of histones as another potential mechanism by which OGT-CDG phenotype is conveyed.

## Histone ubiquitination

The final class of histone modifiers identified in the OGT interactome includes ASXL2, BAP1, and HCFC1 (discussed later) of the PR-DUB complex (Fig. 3) (191). Given that OGT regulates polycomb repression in *Drosophila* (10), it is possible that the PR-DUB complex is essential to that regulation and possibly to the OGT-CDG mechanism in humans. The PR-DUB complex is responsible for removing ubiquitin added to H2A by the Polycomb Repressive Complex 1 (PRC1) (192, 193). ASXL2 has previously been implicated in the neurodevelopmental phenotype (Table 1), but BAP1 has more recently been found causal for the phenotype (Table 1, **bold**). The PR-DUB complex and PRC1 regulate gene expression with both complexes being linked to neurodevelopmental phenotypes (194). BAP1 is known to regulate both OGT and HCFC1 levels by catalyzing their deubiquitination resulting in decreased proteasomal degradation (191). OGT might also regulate levels or the integrity of the PR-DUB complex by stabilizing the complex or by targeting the adaptor protein ASXL2, which can be replaced in the complex by paralogs ASXL1 (MIM#:605036) and ASXL3 (MIM#:615485) both also linked to ID. Of note, OGT-CDG causal variants in the TPR domain all exhibited altered LXR pathway gene expression, and ASXL2 has been shown to increase LXRα activity (32, 195). Moreover, a recent study has shown that BAP1 may play a role as an adaptor protein for OGT (65). Knockout of BAP1 resulted in a small change in global O-GlcNAcylation but had major impacts on the O-GlcNAcylation of ASXL3, HCFC1, and ANKRD17 with HCFC1 having clustered increases and decreases. Lastly, a decrease in global O-GlcNAc levels in *Drosophila* in early development results in redeployment of the PRC, an increase in the H3K27me3 repressive histone modification at genes involved in nervous system development, and a reduction in brain size (196). While this role of O-GlcNAc needs to be confirmed in human systems, this work further supports the notion that OGT and O-GlcNAc are vital for early embryonic development *via* the regulation of histone modifications. In particular histone methylation, acetylation, and ubiquitination could all possibly link OGT-CDG causal variants to the phenotype seen in patients, and further investigation into these histone modifications could elucidate the common perturbations in gene expression seen in the TPR domain variants.

## Nucleosome remodeling

OGT’s interaction with the BAF (mSWI/SNF) complex members SMARCA4, SMARCC2, ARID1A, and ARID1B expands the list of known interactors of OGT (60), and they provide mechanistic targets to understand mechanisms causal in OGT-CDG. The BAF complex, including interactor POGZ, is responsible for remodeling nucleosomes and regulating transcription (Fig. 3) (197, 198, 199, 200, 201, 202, 203, 204, 205, 206). The BAF complex containing SMARCA4 is known to co-regulate nucleosome remodeling (207) as well as the Nucleosome Remodeling Factor (NURF) complex member BPTF (208, 209, 210, 211, 212). POGZ has been studied in relevant models as a high-risk gene for autism, and the altered function of POGZ could explain behavioral problems seen in OGT-CDG patients (197, 213, 214, 215, 216, 217, 218, 219, 220). Furthermore, POGZ is known to co-occupy and coregulate relevant gene expression with ADNP, which was previously identified as a potential conveyor of the OGT-CDG phenotype in a bioinformatic screen (61, 221, 222). Composition and integrity of the BAF complex are essential for early development with the switching of BAF subunits giving rise to embryonic (esBAF), neuroprogenitor (npBAF), and neural (nBAF) specific complexes (Fig. 1*B*) during development (199, 200, 201, 202, 205, 223, 224, 225, 226, 227, 228, 229, 230, 231, 232, 233, 234, 235, 236, 237). Mutually exclusive subunits ARID1A and ARID1B differ greatly in their number of mapped O-GlcNAc sites (22 *versus* 1, respectively). Furthermore, ACTL6A was also identified as an interactor and has more recently been reported to be linked to a neurodevelopmental phenotype (238, 239). Given that ACTL6A is also swapped for ACTL6B (MIM#:618470) during similar developmental time periods as the swap from ARID1A to ARID1B, it is worth investigating any role OGT may play in the composition and integrity of the BAF complex in this neural context. The OGT interactor BPTF is also knocn to regulate neurodevelopment, and its interaction with OGT further highlights the importance of studying nucleosome remodeling in the context of O-GlcNAc biology (208, 209, 210, 240). Given that all OGT-CDG variants in the TPR domain showed altered gene expression and OGT interacts with multiple proteins involved in nucleosome remodeling, it is possible that OGT regulates chromatin accessibility. Using techniques like ATAC-seq to probe chromatin accessibility could provide evidence for the involvement of BAF or other chromatin remodelers in the OGT-CDG phenotype and would elucidate the role OGT plays in chromatin accessibility (32, 241).

## Transcription factors

Beyond epigenetic regulation, the OGT interactome contains several transcription factors, and it has been well established that O-GlcNAc modification of transcription factors can regulate their function (Fig. 3) (26, 242). TAF6 is a part of the TFIID complex required for basal transcription (243, 244, 245, 246, 247) and has links to the SAGA complex mentioned in the section Histone Acetylation (247, 248, 249). The TFIID complex may represent a neurodevelopmentally important node in transcription as TAF1 (MIM#:300966), TAF2 (MIM#:615599), and TAF13 (MIM#:617432) have already been found causal for neurodevelopmental disorders. The OGT interactor TAF4 (60) has recently been associated with a neurodevelopmental disorder expanding the list of OGT interactors and TFIID members causal for ID (250). CIC, the mammalian homolog of Capicua, is a repressor involved in the MAPK signaling pathway (251, 252, 253, 254, 255, 256, 257, 258, 259, 260, 261, 262, 263, 264). The regulation of MAPK signaling has also been previously suggested as a potential effector of the OGT-CDG phenotype (61). Not only is CIC involved in neurodevelopment and function (265, 266, 267, 268, 269, 270), but it also recruits the SIN3A/HDAC and BAF complexes discussed earlier for repression of gene expression (258, 268). The interplay between interactors CIC and SIN3A is one of many known interactions between the proteins discussed herein (Fig. 4) and highlights the complexity and integrated nature of ID causal proteins that interact with OGT. SPEN (MINT/SHARP) is another OGT interactor involved in transcriptional regulation, and it plays a role in the inactivation of the X chromosome (Fig. 3) (271, 272, 273, 274, 275, 276), Notch signaling (277, 278, 279, 280), and Wnt/β-catenin signaling (281, 282). Furthermore, OGT has been shown to regulate neurogenesis in part through Wnt/β-catenin and Notch signaling (283, 284). With 65 O-GlcNAc sites on SPEN (Fig. 2), this interactor should be investigated within the context of O-GlcNAc biology and OGT-CDG as it represents a transcriptional node connecting different signaling pathways important for early development. In particular, O-GlcNAc sites localized to the nuclear receptor interaction domain of SPEN are likely relevant as these sites may regulate protein-protein interactions (65). The OGT interactor and transcription regulator HCFC1 has been hypothesized to play a role in OGT-CDG previously (61, 98, 285). Of the interactors discussed in this review, HCFC1 by far has the most mapped O-GlcNAc sites (Fig. 2) and is the most well-studied in relation to OGT, which suggests that it plays an important role in O-GlcNAc biology. HCFC1 mutations cause ID, but only mutations in the Kelch domain, which lacks O-GlcNAc sites, result in alterations in cobalamin metabolism (286). Pathogenic HCFC1 variants HCFC1^A864T^ (287), HCFC1^G876S^ (288), and HCFC1^A897V^ (289) are proximal to mapped O-GlcNAc sites, suggesting that these sites, and potentially others, are relevant to the neurodevelopmental phenotype seen in OGT-CDG patients. Furthermore, HCFC1 is cleaved by OGT (13, 14, 15, 16, 290, 291), but this function is separable from the glycosylation of HCFC1 (292). Despite the evidence suggesting that HCFC1 glycosylation or cleavage could be linked to the OGT-CDG phenotype, only OGT^N567K^ of the catalytic domain variants shows alterations in glycosylation or cleavage of an HCFC1 repeat substrate *in vivo* (34). However, HCFC1 glycosylation and cleavage by TPR domain variants have not been tested in cells, where protein-protein interactions could affect their function. OGT was found to interact with TET2/3, and through this interaction, TET2/3 promotes the glycosylation of HCFC1 by OGT (293). Furthermore, the glycosylation of HCFC1 favors the integrity of the SET1/COMPASS complex containing SETD1A discussed earlier. Given that TET2 was also identified in the OGT interactome (60), it is possible that cell-type specific interactions with OGT could explain the OGT-CDG phenotype *via* interactors not yet linked to ID. Overall, transcriptional regulators represent important targets of investigation and further add to the evidence that the OGT-CDG mechanism lies in the proper spatiotemporal regulation of gene expression (Fig. 1*B*).

## mRNA processing, export, and silencing

OGT interactors implicated in mRNA processing, export, and silencing further suggest that the OGT-CDG mechanism may involve regulation of gene expression (Fig. 3). With nearly 30 mapped O-GlcNAc sites, ZC3H14 provides a novel target to investigate the role of OGT in mRNA processing, in particular sites S211, S220, and S369 which were found to be enriched in mESCs over that of RA-differentiated mESCs (66). ZC3H14 regulates mRNA processing by controlling poly(A) tail length (294, 295, 296, 297, 298), and its function is important for normal neuronal activity and development (299, 300, 301, 302, 303, 304, 305, 306). NUP62 has been previously identified as a potential target relevant to the OGT-CDG phenotype (285). It is central to the integrity of the Nuclear Pore Complex (NPC), and thus NUP62 plays a role in transport between the nucleus and cytoplasm (307, 308, 309, 310). Without the O-GlcNAc modification on NUP62, it is degraded *via* the proteasome pathway (311). Therefore, it is possible that OGT-CDG patients have lower levels of NPC, resulting in aberrant transport between the nucleus and cytoplasm. Lastly, TNRC6B represents another protein through which OGT could regulate the expression of genes and another link to neurodevelopmental disorders. TNRC6B is a scaffold for the RNA-induced Silencing Complex (RISC) (312, 313, 314, 315, 316, 317, 318, 319), which silences mRNA transcripts post-transcriptionally and has been implicated in normal neuronal function (320, 321). This complex contains other members linked to neurodevelopmental phenotypes, AGO1 (MIM#:620292) and AGO2 (MIM#:619149), and studies could determine what role OGT plays in the regulation of this complex *via* its interaction and modification of TNRC6B. Taken together, OGT interactors ZC3H14, NUP62, and TNRC6B implicate post-transcriptional regulation of gene expression as a critical new area within O-GlcNAc biology and as a possible mechanism by which OGT-CDG is conveyed, namely the alterations in gene expression seen.

## Other mechanistic targets

In this last section, we will discuss both ID-linked interactors that are currently understudied as well as some other potential targets that might be linked to the OGT-CDG mechanism. ANKRD17, PRR12, and AHDC1 represent understudied proteins in the OGT interactome and provide novel targets for O-GlcNAc research. PRR12 has a role in the microRNA-mediated regulation of gliogenesis (322) and has dynamic expression in the developing brain (323). Additionally, placental reduction of OGT in mice results in altered brain microRNA expression (324), but the mechanisms behind this regulation still need to be determined. ANKRD17 has 40 mapped O-GlcNAc sites (Fig. 2) and may regulate YAP1 and the Hippo pathway (325, 326). OGT has been shown to modulate the Hippo pathway (327, 328), but the role ANKRD17 plays in this modulation has yet to be elucidated. Finally, AHDC1 may play a role in DNA methylation during embryogenesis (329). OGT is known to interact with other proteins involved in DNA methylation and demethylation, including the TET family proteins, so studies investigating the interaction between AHDC1 and OGT would further our understanding of OGT’s role in DNA methylation (330). Studies involving ANKRD17, PRR12, and AHDC1 should consider the role that OGT plays in regulating them to better understand their roles in the cell as well as any possible connections to the OGT-CDG phenotype.

This review has focused on those interactors with cataloged ID phenotypes identified using a TPR-BioID approach (60) (Table 1), but as the list has expanded it is worth noting that the OGT-CDG mechanism may involve interactors or substrates not linked to ID yet or discussed in this review. For example, TET2 was discussed in a previous section as it was related to the modulation of HCFC1 O-GlcNAcylation (293). Beyond being identified as an OGT interactor (60), TET2 has been shown to interact with OGT in mESCs (331), has O-GlcNAc sites enriched in mESCs compared to differentiated mESCs (66), and its modification by OGT competes with known phosphorylation sites (332). While TET2 has not been linked to an ID phenotype, studies have shown that TET2 plays a role in neuronal differentiation (333, 334, 335). Quantification of methylated and hydroxymethylated DNA would provide insight into any role of TET2 in the OGT-CDG mechanism (336). Furthermore, numerous heterogeneous nuclear ribonulceoproteins (HNRNP) were identified as OGT interactors in another study (65), and this particular family of proteins has been identified as a hub for shared neurodevelopmental disorders (337). This family of proteins is responsible for multiple aspects of nucleic acid metabolism including alternative splicing and transcriptional regulation (338), which were discussed in this review as possible mechanisms responsible for OGT-CDG. While they were not discussed in detail, they further highlight the need for the field to investigate the role of O-GlcNAc biology in the regulation of gene expression especially as it relates to possible OGT-CDG mechanisms. While other avenues of investigation will be necessary to fully understand the OGT-CDG phenotype, the large number of OGT interactors and substrates discussed herein highlights the need to probe the role of transcription regulation in the OGT-CDG phenotype.

## Future directions

Since the first characterizations of OGT-CDG causal mutations (30, 31), the list of OGT variants has expanded (Table 1) and continues to expand as clinicians test for the genetic causes of ID in patients. In this review, we have discussed the variable biochemical characteristics of the OGT-CDG variants, with defects in glycosylation representing the common alteration though to different degrees depending on the domain. The variable nature of the patient phenotype has been highlighted, and we discussed how animal and cell models recapitulate this variability. We have also provided evidence that, in part, the OGT-CDG mechanism lies in the dysregulation of gene expression as shown by models of TPR domain variants in hESCs and a catalytic domain variant in mESCs (32, 34). This common downstream impact of the variants led to the evaluation of the OGT interactome, which is enriched for gene expression regulators causal for ID phenotypes. Beyond investigating these interactors for links to the OGT-CDG mechanisms, there are other pertinent questions that remain. Could supplementation with glucosamine represent a therapeutic for patients as previously suggested (98)? If models can show that glucosamine supplementation can alleviate the observed effects of OGT-CDG variants, then the next step would be for clinicians to see if supplementation could alleviate some symptoms as has been tested for other CDGs (339). TPR domain variants should also be tested for any alterations in their interactomes in relevant cell types to further narrow the list of likely mechanistic targets. Additionally, the gene expression changes seen in patient lymphoblastoids has not been validated for other variants, but if it is seen in other patients, RNA-seq may represent a diagnostic tool for clinicians to rapidly identify OGT-CDG patients if a set of commonly altered genes is identified. Moreover, models have shown that OGT-CDG variants attempt to maintain O-GlcNAc homeostasis and OGA is essential for normal embryogenesis (340), which elicits the question of whether mutations in OGA might also be causal for an ID phenotype yet to be discovered. Finally, the studies to date show varied effects in models. For example, OGT^C921Y^ shows defects in maintaining the pluripotency network, but other variants do not. Do these varied effects represent differences in study design or do they suggest that OGT-CDG variants may have different mechanistic targets that are affected? Future studies would benefit from the use of multiple OGT-CDG variants to uncover shared or unique affected targets to better address this question. While many questions remain for us to fully understand OGT-CDG, many have been answered since the first characterization of variants, and studies utilizing OGT-CDG variants give us further insight into OGT and O-GlcNAc biology.

## Conflict of interest

The authors declare that they have no conflicts of interest with the contents of this article.
